# Epidemiological characteristics and whole-genome analysis of respiratory syncytial virus in Jining city from February 2023 to December 2024

**DOI:** 10.3389/fmicb.2026.1702525

**Published:** 2026-02-11

**Authors:** Julong Wu, Yongjian Jia, Yajuan Jiang, Feifei He, Xuezhen Shi, Xiaoyu Wang, Ying Yue, Wei Liu, Huixin Dou, Boyan Jiao

**Affiliations:** 1 Department of Infectious Disease Control, Shandong Center for Disease Control and Prevention, Jinan, China; 2 Shandong Provincial Key Laboratory of Intelligent Monitoring, Early Warning, Prevention and Control for Infectious Diseases, Jinan, China; 3 Department of Laboratory, Jining Center for Disease Control and Prevention, Jining, China; 4 Jining Key Laboratory of Infectious Disease Control and Prevention, Jining, China; 5 Computer Information Technology, Northern Arizona University, Flagstaff, AZ, United States; 6 Gynecology Department, Baoding First Central Hospital, Baoding, China; 7 Department of Infectious Disease Control, Jining Center for Disease Control and Prevention, Jining, China; 8 Director's Office, Jining Center for Disease Control and Prevention, Jining, China

**Keywords:** antigenic variation, epidemiology, phylogenetics, respiratory syncytial virus, whole-genome sequencing

## Abstract

**Background:**

Respiratory syncytial virus (RSV) is a major viral pathogen causing respiratory tract infections in children, often leading to bronchiolitis, pneumonia, and even death. This study aimed to investigate the epidemiological patterns and whole-genome characteristics of RSV circulating in Jining city between February 2023 and December 2024.

**Methods:**

From February 2023 to December 2024, a total of 5,042 throat swab samples were collected from influenza-like illness (ILI) cases at two sentinel hospitals in Jining. RSV was detected using reverse transcription quantitative real-time PCR (RT-qPCR). RSV-positive samples were subjected to whole-genome sequencing. Phylogenetic trees were constructed based on the whole genome and G gene sequences, and antigenic variation in viral proteins was analyzed.

**Results:**

RSV positivity was 1.98% (100/5042), with higher rates in children under 5 years of age. RSV activity peaked in April–May 2023 and December 2023–January 2024. A total of 29 RSV genomes were sequenced, including 18 RSV-A and 11 RSV-B. RSV-A was dominant during the first peak, and RSV-B during the second. Most RSV-A strains belonged to clade AD.3 and genotype ON1; RSV-B strains clustered into clades B.D.E.1, B.D.4.1.1, and B.D.E.2, all within genotype BA9. Mutations were identified in antigenic epitopes of the G and F proteins, including amino acid substitutions and changes in glycosylation and phosphorylation sites.

**Conclusion:**

RSV-A and RSV-B circulated in Jining in an alternating pattern, with evidence of ongoing antigenic evolution. Continued RSV surveillance and accelerated vaccine development are essential.

## 1 Introduction

Respiratory syncytial virus (RSV), a membrane-enveloped RNA virus belonging to the genus *Orthopneumovirus* within the family *Paramyxoviridae*, exists in both spherical (10–350 nm) and filamentous (60–200 nm) forms (Duan et al., 2024; Sanz-Munoz et al., 2024; Jeffree et al., 2003). RSV infection can cause a wide range of clinical symptoms, including fever, rhinorrhea, cough, dyspnea, wheezing, bronchiolitis, pneumonia, and even death (Chaiut et al., 2023; Li et al., 2023). Globally, RSV is responsible for an estimated 33 million cases, 3.6 million hospitalizations, and approximately 100,000 deaths annually, representing a significant disease burden (Li et al., 2022).

RSV encodes several structural and non-structural proteins, including NS1, NS2, N, P, M, SH, G, F, M2-1, M2-2, and L. Among these, the G (attachment) and F (fusion) glycoproteins play critical roles in viral entry and host cell interaction and contain major antigenic epitopes (Duan et al., 2024; Sanz-Munoz et al., 2024). Based on antigenic and genetic variability in the G gene, RSV is classified into two antigenic subtypes: RSV-A and RSV-B (Anderson et al., 1985; Sullender, 2000). Further genotyping based on the G gene sequences has led to the identification of multiple genotypes: GA1–GA7, SAA1, NA1–NA4, and ON1–ON2 for RSV-A; and BA1–BA14, GB1–GB5, SAB1–SAB4, URU1–URU2, NZB1–NZB2, BA-CCA, BA-CCB, BA-C, CBB, and CB1 for RSV-B (Sanz-Munoz et al., 2024; Peret et al., 1998; Bashir et al., 2015). With the development of next-generation sequencing (NGS), whole-genome sequencing has been increasingly applied to study RSV evolution and clade classification at a higher resolution (Pangesti et al., 2018; Dong et al., 2025; Wei et al., 2024; Sondlane et al., 2024; Tramuto et al., 2024).

In China, RSV is one of the leading causes of respiratory illness, with significant regional and seasonal variation in prevalence. Typically, RSV circulates during the winter and spring seasons, although outbreaks have also been observed in early summer in certain years. RSV-A and RSV-B often alternate in dominance between epidemic seasons, with RSV-A generally being more prevalent (Li B. S. et al., 2024; Cui et al., 2024).

Jining is a major city in northern China with a population exceeding 8.2 million (Yin et al., 2024; Jiang et al., 2024). As a regional hub, it plays an important role in infectious disease surveillance and has been the focus of several viral epidemiology studies (Wu et al., 2024; Fu et al., 2025; Dou et al., 2025). In this study, we analyzed the epidemiological characteristics of RSV in Jining from February 2023 to December 2024 and performed evolutionary analyses on 29 RSV whole-genome sequences. The findings provide important insights into RSV circulation and molecular evolution in this region, contributing valuable data for RSV surveillance and early warning systems.

## 2 Materials and methods

### 2.1 Study participants

From February 2023 to December 2024, throat swab samples were collected within 3 days of symptom onset from patients with influenza-like illness (defined as fever ≥38 °C accompanied by cough or sore throat) at Jining No.1 People’s Hospital and Rencheng District Maternal and Child Health Hospital. Swabs were placed in viral transport medium containing RNase inhibitor produced by Youkang Biotechnology Co., Ltd. (Beijing, China; catalog no. MT0901). A total of 5,042 samples were collected, including 4,450 from outpatients and 592 from hospitalized patients.

### 2.2 Nucleic acid detection

A 200 μL aliquot of each throat swab sample was used for nucleic acid extraction, performed using the SSNP-9600A automated extraction system and purification kit (catalog no. SDKF60101) from Jiangsu Shuoshi Biotechnology Co., Ltd. RSV RNA was detected using a fluorescent RT-qPCR kit (catalog no. SKY-8237) from Shenzhen Shengkeyuan Biotechnology Co., Ltd. with the following cycling conditions: 50 °C for 15 min; 95 °C for 3 min; followed by 45 cycles of 95 °C for 5 s and 55 °C for 30 s. RSV subtype (A/B) was determined using a dual detection RT-qPCR kit (catalog no. SKY-8240) from the same manufacturer under identical cycling conditions. Prior to use, the laboratory performed performance verification of the reagents to determine the limit of detection (LOD). In each experimental run, negative and positive quality control samples were included, and the human RNase P endogenous reference gene was detected concurrently. To ensure the accuracy and reliability of the detection results, the viral transport medium (VTM) utilized was a virus preservation solution containing an RNase inhibitor. Additionally, LOD performance verification of the reagents was conducted by the laboratory before use, with negative and positive quality control samples incorporated in every run and the human RNase P internal reference gene monitored throughout.

### 2.3 Whole-genome sequencing

Whole-genome sequencing was performed on 29 RSV-positive samples with Ct values ≤30. Among the 29 cases, 16 were male and 13 female; age distribution was as follows: 17 aged 1–<5 years, 6 aged 5–<15 years, 3 aged 25–<60 years, and 3 aged ≥60 years; 21 samples were collected in 2023 and 8 in 2024.

Reverse transcription was performed using the RSV Whole-Genome Capture Kit (catalog no. BK-RSVAB024) from Hangzhou Boyi Technology Co., Ltd. with a 15 μL reaction mix (3 μL RT Super Mix, 12 μL RNA template). Conditions: 25 °C for 10 min, 50 °C for 10 min, 85 °C for 5 min, 4 °C hold. Amplification was conducted with 2.5 μL RT product, 12.5 μL HotStart HiFi Mix, 4 μL primer pool (A1/A2/B1/B2), and 6 μL nuclease-free water. Cycling conditions: 98 °C for 30 s; 5 cycles of 98 °C for 15 s and 63 °C for 5 min; followed by 25–30 cycles of 98 °C for 15 s and 65 °C for 4 min; final extension at 72 °C for 2 min.

Purification was performed using AMPure XP beads (Beckman Coulter, catalog no. A63880), and quantification was done using a Qubit 3 fluorometer (Invitrogen). cDNA fragmentation was conducted using the Nextera XT DNA Library Preparation Kit (Illumina, catalog no. 15032350), and adapter ligation was carried out using Nextera XT Index Kit v2 Set A (catalog no. 15052163) with the following cycling conditions: 72 °C for 3 min, 95 °C for 30 s; then 12 cycles of 95 °C for 10 s, 55 °C for 30 s, and 72 °C for 30 s; final extension at 72 °C for 5 min. Libraries were sequenced using the Illumina NextSeq 2000 platform with a 300-cycle NextSeqTM 1000/2000 P1 reagent cartridge (catalog no. 20049920).

### 2.4 Sequence alignment and analysis

Sequence assembly and analysis were performed using CLC Genomics Workbench 24 (QIAGEN). RSV whole-genome sequences were aligned and compared using BLAST against reference sequences in the NCBI database. Representative early ON1 and BA9 strains from China—RSV-A isolate BJ/40180 (GenBank: MF614946.1) and RSV-B strain RSVB/BCH-Y/2016 (GenBank: KY924878.1)—as well as recent RSV genomes were downloaded from GenBank. Multiple sequence alignments were performed using MEGA 7.0.14, and maximum-likelihood phylogenetic trees were constructed with 1,000 bootstrap and Tamura-Nei model replicates based on the whole genome and G gene sequences. Similarity analysis of RSV genes and encoded proteins was performed using MEGA 7.0.14 and MegAlign (DNASTAR 7.0.1). G protein amino acid variations were analyzed using MEGA 7.0.14.

### 2.5 Statistical analysis

All statistical analyses were conducted using SPSS version 25.0. Group comparisons were performed using the chi-square (χ^2^) test. A *p*-value < 0.05 was considered statistically significant.

## 3 Results

### 3.1 Epidemiological characteristics of RSV

Among the 5,042 influenza-like illness (ILI) samples collected from February 2023 to December 2024, Among these, 4,450 cases were outpatients and 592 were inpatients.

**Figure 1:**
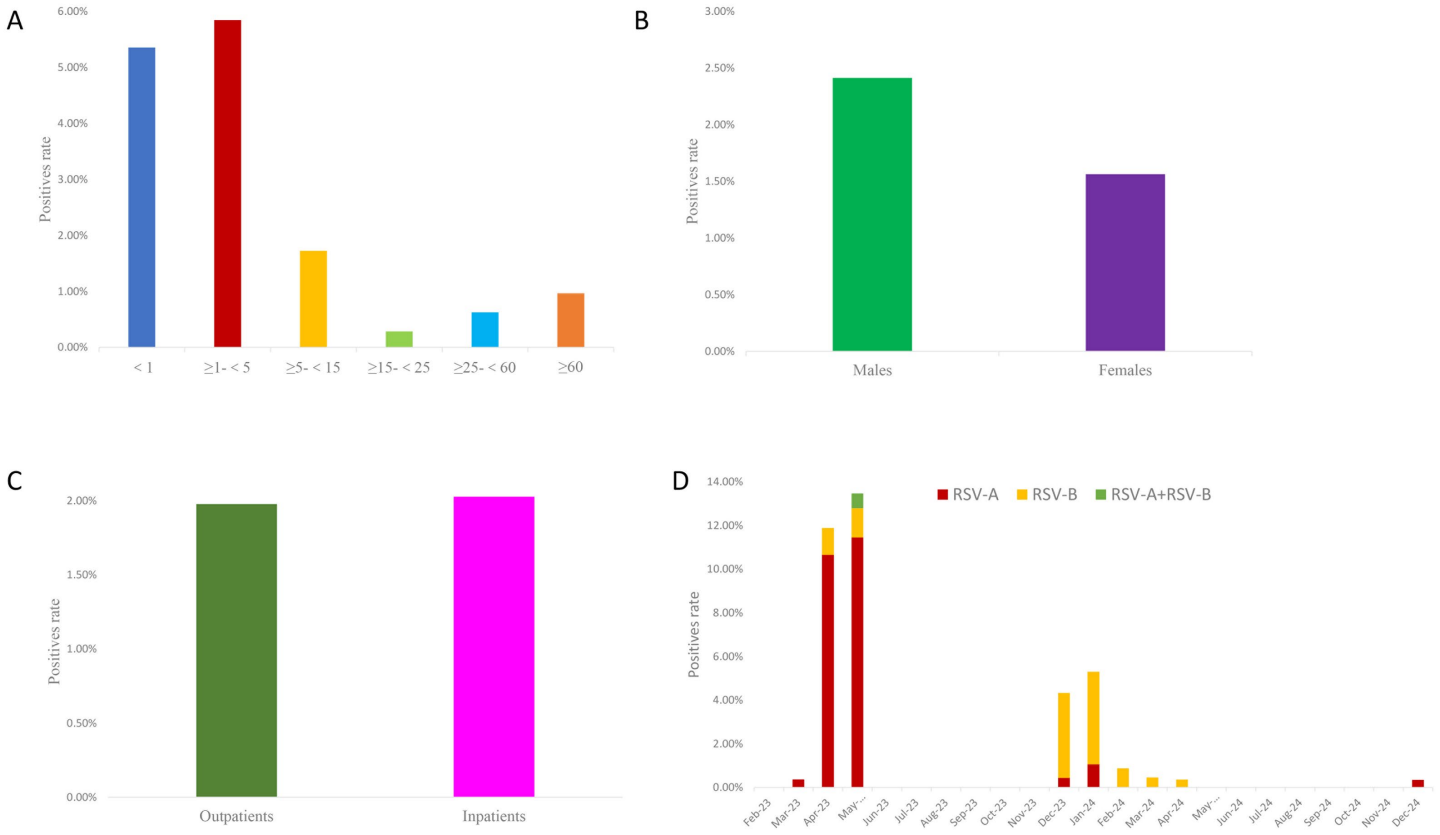
Epidemiological characteristics of RSV in Jining, China (February 2023–December 2024). **(A)** RSV positivity rates across different age groups. The *x*-axis represents age groups, and the *y*-axis indicates positivity rates (%). **(B)** RSV positivity rates by sex. The *x*-axis represents sex (male vs. female), and the *y*-axis indicates positivity rates (%). **(C)** RSV positivity rates among inpatients and outpatients. The *x*-axis represents patient setting (inpatient vs. outpatient), and the *y*-axis indicates positivity rates (%). **(D)** Monthly distribution of RSV positivity rates. The *x*-axis represents calendar months, and the *y*-axis indicates positivity rates (%).

100 tested positive for RSV, yielding a positivity rate of 1.98% (100/5042). The infection rate was highest among children under 1 year and those aged 1 to <5 years, while lower rates were observed in the 15–<25 and 25–<60 age groups. The differences in RSV positivity across age groups were statistically significant (χ^2^ = 108.53, *p* < 0.001). The RSV positivity rate was higher in males (2.41%, 60/2485) compared to females (1.56%, 40/2557), and the gender difference was statistically significant (χ^2^ = 4.685, *p* = 0.03). However, no significant difference was observed between outpatients and inpatients (χ^2^ = 0.01, *p* = 0.93) (Supplementary Table 1; Figure 1).

Seasonal variation in RSV activity was evident. Two distinct peaks were observed: one in April–May 2023 and another in December 2023–January 2024, during which RSV positivity rates were significantly higher compared to other months (χ^2^ = 74.68, *p* < 0.001). RSV-A was the predominant subtype during the April–May 2023 peak, whereas RSV-B dominated in December 2023–January 2024. The difference in the proportion of predominant strains between the April–May 2023 peak and the December 2023–January 2024 peak was statistically significant (χ^2^ = 50.22, *p* < 0.001) (Supplementary Table 2; Figure 1). In addition to RSV testing, all 5,042 influenza-like illness (ILI) samples were also screened for influenza A (H1N1), H3N2 influenza virus, influenza B virus, SARS-CoV-2, and human adenovirus (HAdV). The positivity rates were as follows: influenza A (H1N1), 6.27% (316/5,042); H3N2 influenza virus, 9.26% (467/5,042); influenza B virus, 3.91% (197/5,042); SARS-CoV-2, 12.65% (638/5,042); and HAdV, 3.55% (179/5,042). To provide context for the low RSV positivity rate and to rule out co-circulation of other common respiratory viruses, all 5,042 ILI samples were also tested for influenza A (H1N1), H3N2, influenza B, SARS-CoV-2, and human adenovirus (HAdV). The positivity rates were 6.27% (316/5,042), 9.26% (467/5,042), 3.91% (197/5,042), 12.65% (638/5,042), and 3.55% (179/5,042), respectively. Detailed individual-level data, including gender, age, outpatient/inpatient status, sampling date, and detection results for all tested pathogens, are provided in Supplementary Table S2.

### 3.2 Whole-genome sequencing of RSV

Whole-genome sequencing was successfully performed on 29 RSV-positive samples, yielding complete genomes ranging from 14,590 to 15,221 base pairs in length. Among them, 18 sequences were classified as RSV-A and 11 as RSV-B. Of the 21 sequences obtained in 2023, 16 were RSV-A and 5 were RSV-B. In contrast, the 8 sequences from 2024 included 2 RSV-A and 6 RSV-B strains. The chi-square (χ^2^) test was used to compare the 29 sequenced RSV-positive samples with all 100 RSV-positive samples in terms of time period, sex, and age. The results showed no significant differences for time period (χ^2^ = 0.76, *p* = 0.38), sex (χ^2^ = 0.21, *p* = 0.64), or age (χ^2^ = 1.67, *p* = 0.89), indicating that the 29 sequenced RSV-positive samples were highly representative of the overall cohort. All RSV-A strains belonged to the ON1 genotype, and all RSV-B strains were classified as BA9 genotype. Amino acid variations in key proteins were analyzed using linear mutation maps (Supplementary Figure 1 for RSV-A; Supplementary Figure 2 for RSV-B). Structural predictions further illustrated the Q311stop mutation in the G protein C-terminus (Supplementary Figure 3) and key substitutions (K191R, I206M, Q209R, S389P, S190N) in the F protein (Supplementary Figure 4).

### 3.3 Sequence similarity analysis

**Figure 2:**
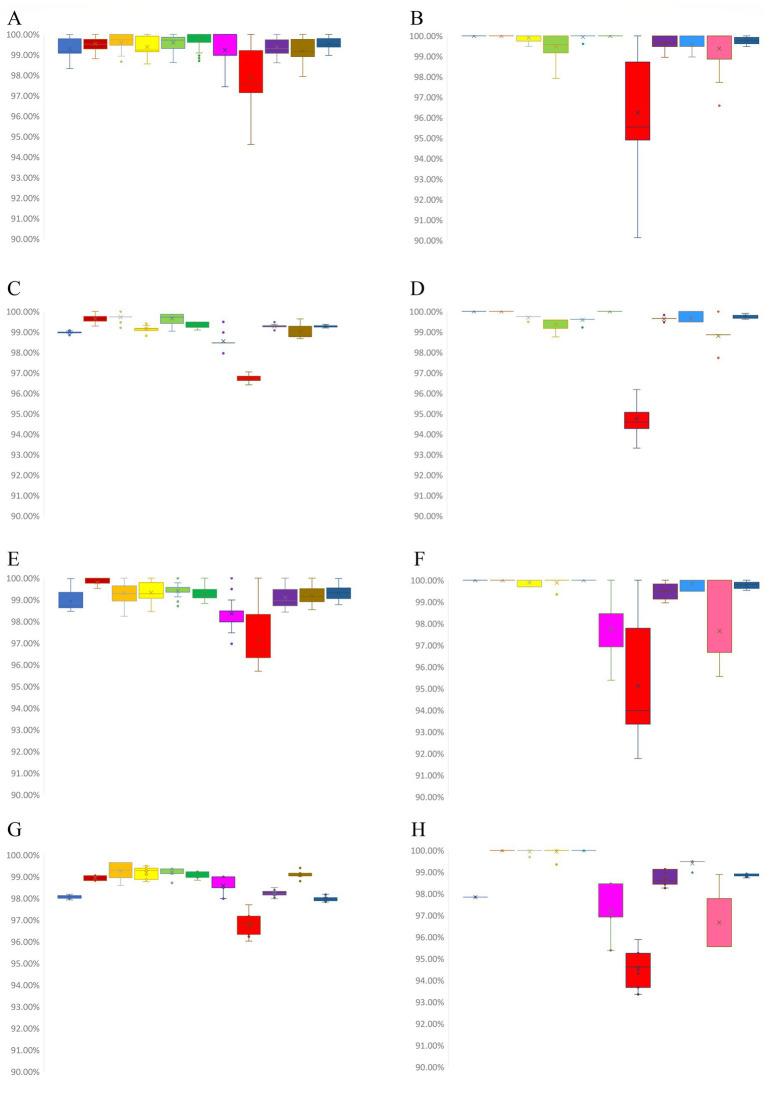
Similarity analysis of RSV genomes and encoded proteins. **(A)** Nucleotide similarity among the 18 RSV-A ON1 genomes from Jining. **(B)** Amino acid similarity of encoded proteins among the 18 RSV-A ON1 sequences from Jining. **(C)** Nucleotide similarity between the 18 RSV-A ON1 genomes from Jining and the early Chinese ON1 reference strain BJ/40180. **(D)** Amino acid similarity of encoded proteins between the 18 RSV-A ON1 sequences from Jining and BJ/40180. **(E)** Nucleotide similarity among the 11 RSV-B BA9 genomes from Jining. **(F)** Amino acid similarity of encoded proteins among the 11 RSV-B BA9 sequences from Jining. **(G)** Nucleotide similarity between the 11 RSV-B BA9 genomes from Jining and the early Chinese BA9 reference strain RSVB/BCH-Y/2016. **(H)** Amino acid similarity of encoded proteins between the 11 RSV-B BA9 sequences from Jining and RSVB/BCH-Y/2016.

Among the 18 ON1 genotype RSV-A sequences from Jining, whole-genome nucleotide identity ranged from 98.33 to 99.99%. The nucleotide similarity for the G and F genes ranged from 94.61 to 100% and 98.61 to 100%, respectively. At the amino acid level, the G and F proteins shared 90.13–100% and 98.95–100% identity, respectively. Compared with an earlier Chinese ON1 strain, RSV-A isolate BJ/40180 (GenBank: MF614946.1) (Cui et al., 2017), whole-genome nucleotide identity ranged from 98.84 to 99.07%, G and F gene nucleotide identity ranged from 96.41 to 97.04% and 99.07 to 99.48%, and G and F protein amino acid identity ranged from 93.31 to 96.18% and 99.48 to 99.83%, respectively (Supplementary Table 3; Figures 2A–D).

For the 11 BA9 genotype RSV-B sequences, whole-genome nucleotide similarity ranged from 98.46 to 99.98%. The G and F gene nucleotide identity ranged from 95.70 to 100% and 98.43 to 100%, while the G and F protein amino acid identity ranged from 91.77 to 100% and 98.95 to 100%, respectively. Compared with the early Chinese BA9 reference strain RSVB/BCH-Y/2016 (Xu et al., 2018), whole-genome nucleotide identity ranged from 97.92 to 98.18%. G and F gene nucleotide identity ranged from 96.02 to 97.69% and 97.97 to 98.49%, while G and F protein amino acid identity ranged from 93.35 to 95.89% and 98.26 to 99.13%, respectively (Supplementary Table 4; Figures 2E–H).

### 3.4 Phylogenetic analysis

**Figure 3:**
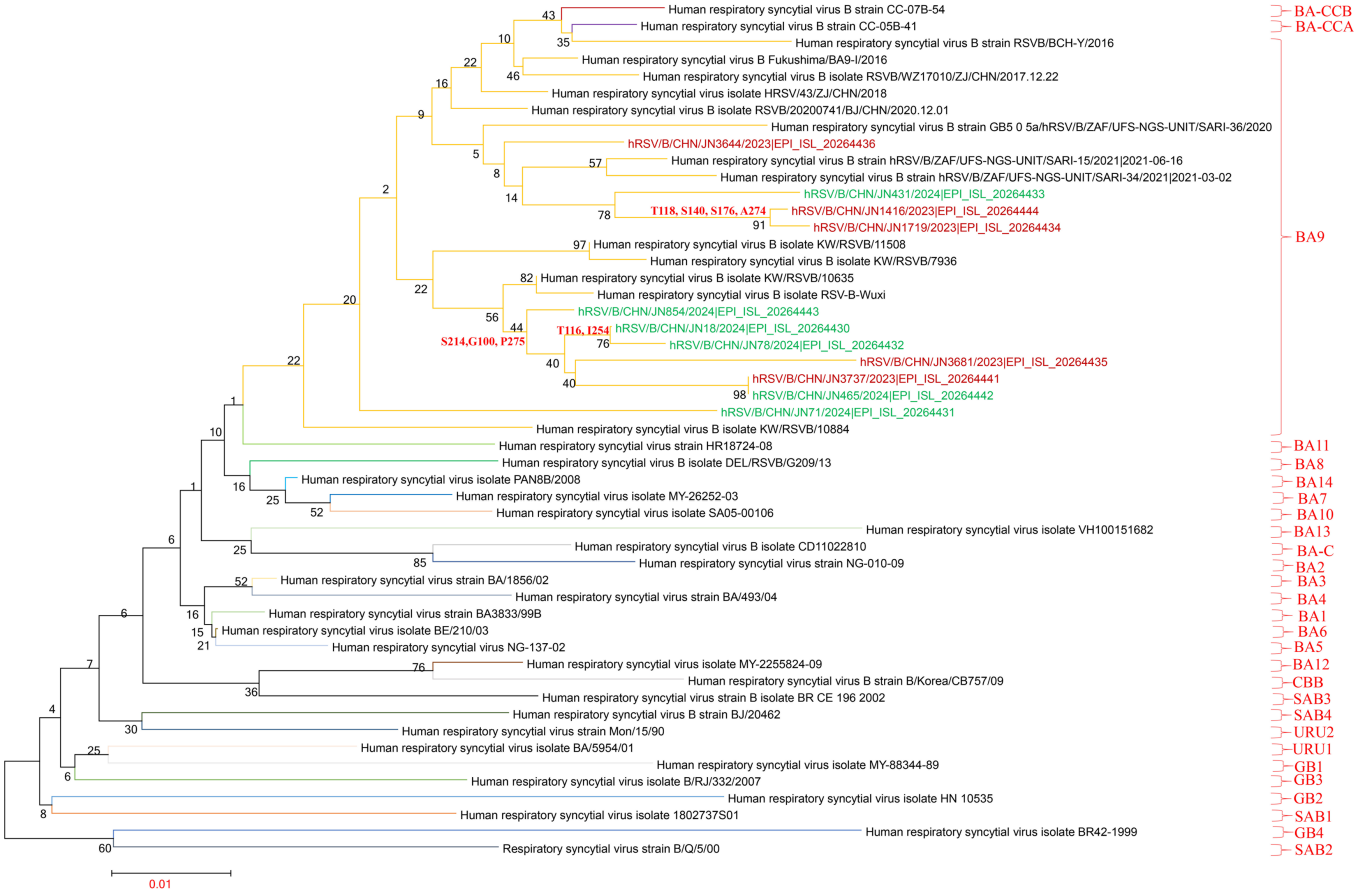
Phylogenetic analysis of RSV-A G gene sequences. The phylogenetic tree was constructed using MEGA version 7.0.14 with the maximum-likelihood method and 1,000 bootstrap replicates. Brown branches represent RSV-A G gene sequences from Jining collected in 2023; green branches represent sequences collected in 2024.

Phylogenetic analysis of the RSV-A G gene was performed using 18 sequences obtained from Jining, along with representative sequences of globally circulating RSV-A genotypes from recent years. All 18 Jining strains clustered within the ON1 lineage, confirming their classification as ON1 genotype. Notably, these sequences were distributed across six distinct sublineages within the ON1 clade, suggesting substantial intra-genotypic diversification. This finding indicates that multiple ON1 variants in Jining during the study period, contributing to local RSV-A transmission (Figure 3; Supplementary Table 5).

**Figure 4:**
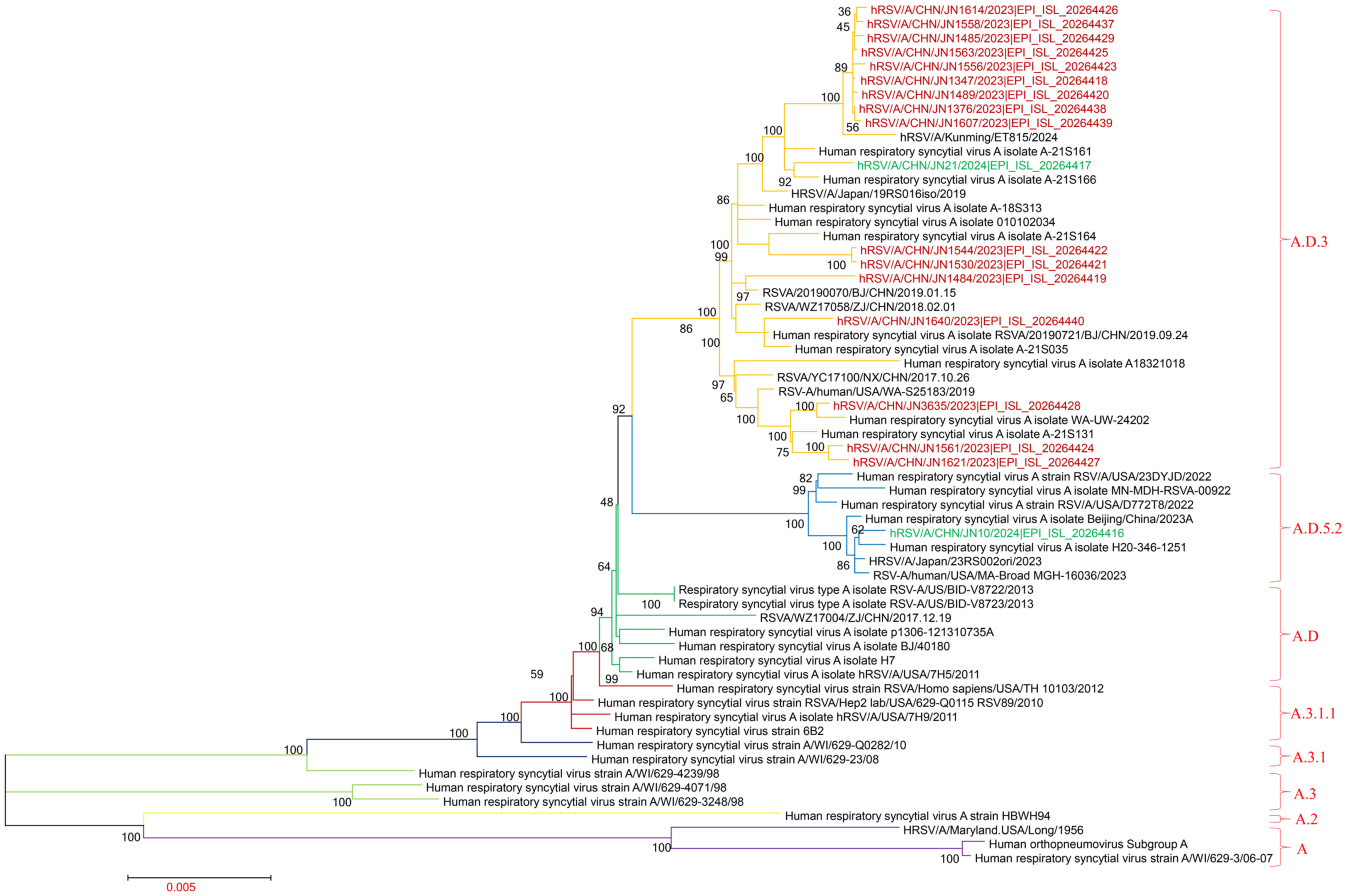
Phylogenetic analysis of RSV-B G gene sequences. The phylogenetic tree was constructed using MEGA version 7.0.14 with the maximum-likelihood method and 1,000 bootstrap replicates. Brown branches represent RSV-B G gene sequences from Jining collected in 2023; green branches represent sequences collected in 2024.

To further characterize RSV-A evolutionary dynamics, whole-genome clade assignments were determined using the Nextstrain platform.^1^ Of the 18 RSV-A genomes, 17 were classified as clade A.D.3 and one as A.D.5.2. These results were corroborated by a maximum-likelihood phylogenetic tree constructed from whole-genome sequences, which yielded identical clade groupings (Figure 4; Supplementary Table 5).

**Figure 5:**
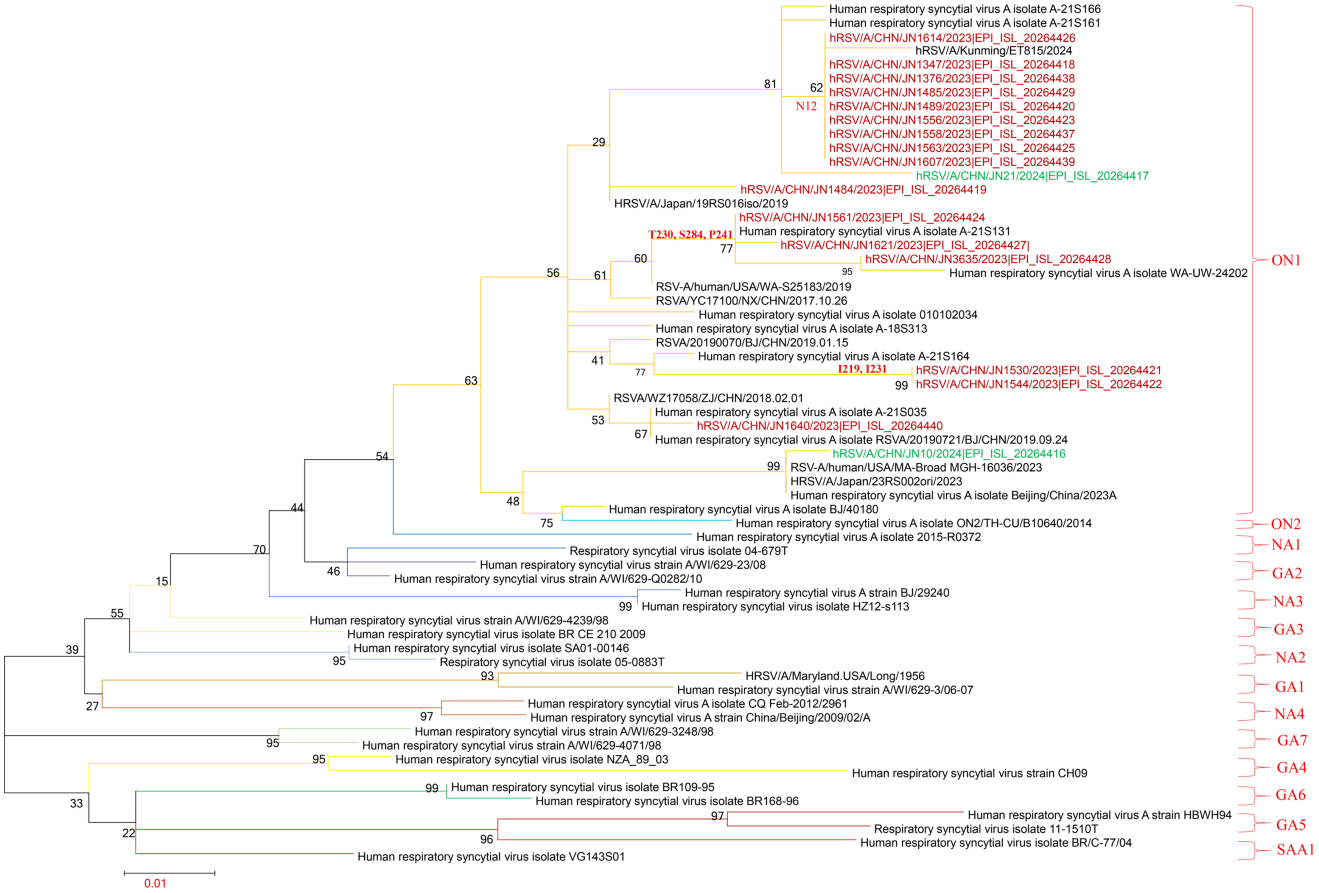
Phylogenetic analysis of complete RSV-A genomes. The phylogenetic tree was constructed using MEGA version 7.0.14 with the maximum-likelihood method and 1,000 bootstrap replicates. Brown branches represent RSV-A complete genome sequences from Jining collected in 2023; green branches represent sequences collected in 2024.

Similarly, phylogenetic analysis of the G gene from 11 RSV-B strains revealed that all isolates clustered within the BA9 lineage, indicating a single prevailing genotype in Jining. However, the sequences fell into three distinct subclusters within the BA9 clade, reflecting ongoing genetic diversification among local RSV-B strains (Figure 5; Supplementary Table 5).

**Figure 6:**
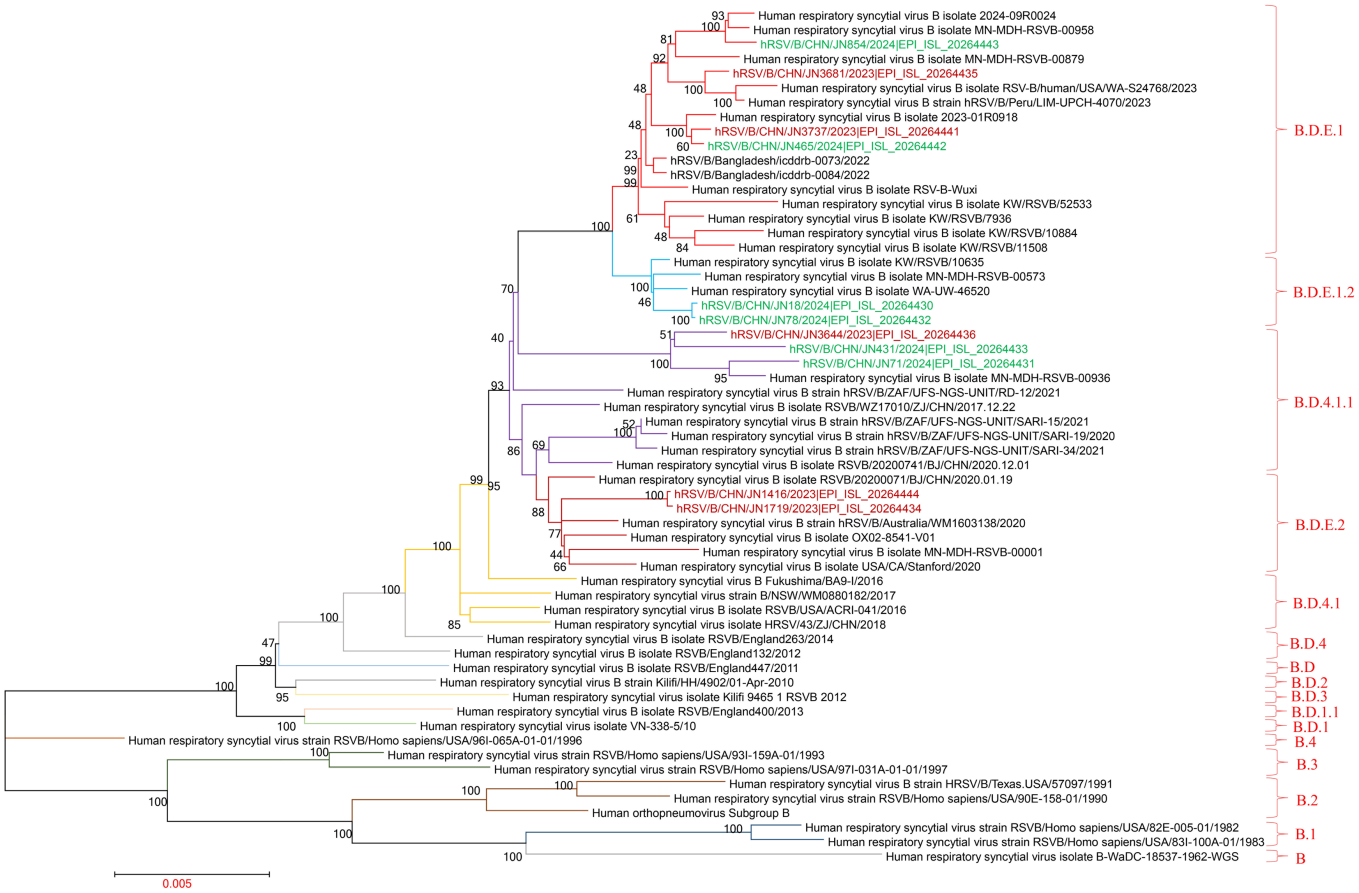
Phylogenetic analysis of complete RSV-B genomes. The phylogenetic tree was constructed using MEGA version 7.0.14 with the maximum-likelihood method and 1,000 bootstrap replicates. Brown branches represent RSV-B complete genome sequences from Jining collected in 2023; green branches represent sequences collected in 2024.

Clade classification of the RSV-B whole genomes using the Nextstrain platform showed that four sequences belonged to clade B.D.E.1, two to B.D.E.1.2, three to B.D.4.1.1, and two to B.D.E.2. These assignments were consistent with results from whole-genome phylogenetic reconstruction, which also placed six sequences in B.D.E.1, three in B.D.4.1.1, and two in B.D.E.2 (Figure 6; Supplementary Table 5).

### 3.5 Amino acid variation analysis

Comparative analysis with the early Chinese ON1 reference strain BJ/40180 revealed that all 18 RSV-A ON1 genomes from Jining harbored amino acid substitutions in multiple proteins, including N, P, M, G, F, M2-1, M2-2, and L.

The G glycoprotein, which mediates host cell attachment and monoclonal antibody recognition, contains a conserved CX3C motif responsible for binding to the host receptor CX3CR1 and facilitating viral adhesion (Duan et al., 2024; Sanz-Munoz et al., 2024). This motif remained intact in all 18 Jining ON1 strains. The G and F proteins are the primary targets for virus-neutralizing antibodies (Huang et al., 2019). Critical antigenic epitopes of the G protein are located at residues 153–197 and the C-terminal 84–85 residues (Jones et al., 2018; Cane et al., 1996; Anderson et al., 2021). Among the ON1 sequences, a single strain carried an N178G mutation within the 153–197 region. However, extensive variability was observed in the C-terminal region, where 28 unique amino acid substitutions were identified (Table 1).

**Table 1:** Amino acid substitutions in RSV-A proteins among 18 ON1 strains from Jining compared to the early Chinese ON1 reference strain BJ/40180.

Protein	Amino acid substitution sites compared with BJ/40180
NS1	-
NS2	-
N	I104V (5.56%), N105S (11.11%), S372A (100%)
P	E49D (5.56%), I66V (5.56%), T69I (5.56%), T72N (16.67%), E89D (5.56%), T92M (94.44%), R191K (16.67%), E239D (5.56%)
M	N27D (100%), M73L (5.56%)
SH	-
G	A10T (5.56%), T12N (50.00%), R15K (11.11%), A57V (5.56%), L71P (5.56%), I76T (50.00%), S88P (100%), P95S (22.22%), G98E (11.11%), F101I (11.11%), S102T (11.11%), T113I (94.44%), I114T (11.11%), P115L (100%), A116D (16.67%), E123K (5.56%), S124P (5.56%), V131D (94.44%), I133T (5.56%), Q140R (5.56%), P143S (5.56%), K145R (5.56%), N178G (5.56%), P206Q (5.56%), K209R (11.11%), T219I (11.11%), V225A (55.56%), V225I (5.56%), L226I (11.11%), P230T (16.67%), T231I (11.11%), P234S (5.56%), T241P (16.67%), T245A (5.56%), L248F (55.56%), L248I (5.56%), G254R (5.56%), H258Q (83.33%), H258L (11.11%), S260G (5.56%), H266L (94.44%), E271K (11.11%), Y273H (55.56%), L274P (66.67%), P276L (11.11%), S283P (5.56%), G284S (16.67%), G284D (5.56%), L289I (11.11%), L289P (5.56%), S291L (5.56%), Y297H (5.56%), L298P (61.11%), S299N (66.67%), V303A (5.56%), Y304H (5.56%), K308E (100%), L310P (61.11%), T309I (55.56%), T320A (5.56%)
F	T12I (94.44%), L15F (5.56%), A23T (5.56%), A103T (11.11%), R113K (5.56%), Y117H (5.56%), N121S (50.00%), T122A (5.56%), I221L (5.56%), S276N (16.67%), Q501K (5.56%)
M2-1	K113R (11.11%), H187N (50.00%), A188V (5.56%)
M2-2	P2S (5.56%), C24Y (5.56%), S60T (5.56%), T77A (88.89%)
L	G7E (5.56%), K76R (5.56%), V151A (11.11%), V151I (11.11%), A173S (16.67%), L835M (94.44%), R1186K (11.11%), R1189K (11.11%), I1191V (11.11%), L1395I (5.56%), V1398I (11.11%), G1550S (22.22%), D1644E (55.56%), S1723G (5.56%), E1725G (100%), N1737T (50.00%), I1741M (11.11%), S1745T (5.56%), I1780V (5.56%), H1798Q (5.56%), I1917T (11.11%), T1942I (5.56%), V1943D (5.56%), T1969I (5.56%), H2135Y (11.11%)

The F protein comprises six major antigenic sites: site Ø (aa 62–69, 196–209), site I (aa 380–400), site II (aa 255–276), site III (aa 46–54, 305–310), site IV (aa 427–437), and site V (aa 148–194) (Huang et al., 2019). Three ON1 strains exhibited an S276N substitution located in site II. Within the p27 domain (aa 110–136), which is critical for membrane fusion (Neal et al., 2024), observed mutations included R113K, Y117H, N121S (in 9 strains), and T122A (Table 1).

The L, P, N, and M2-1 proteins constitute the ribonucleoprotein (RNP) complex, which is essential for viral RNA transcription and replication (Cao et al., 2021; Collins et al., 1996). All known functional residues of M2-1 required for RNA binding (S58, S61, K92, K150, R151, K159) and interaction with P (R126, T130, Y134, S137, Q144, R151, P153, T160, N163) remained fully conserved (Tanner et al., 2014; Gao et al., 2020). One ON1 strain carried an E239D substitution in the P protein, located at a critical interface for N protein binding (Tran et al., 2007), which may affect RNP complex formation. Residues in the N protein mediating binding to P (R259, L272, Y288, F300, F320) and to viral RNA (K170, D175, R184, R185) were conserved in all sequences (Esneau et al., 2019). The L protein contains three enzymatic domains—RNA-dependent RNA polymerase (RdRp), methyltransferase (Mtase), and polyribonucleotidyltransferase (PRNTase). Catalytic residues for RdRp (D700, G810, D811, N812) (Shirai et al., 2022; Noton et al., 2014; Sutto-Ortiz et al., 2023), Mtase (K1831, D1936, K1973, E2004) (Sutto-Ortiz et al., 2021), and PRNTase (G1264, T1267, H1338, R1339) (Gilman et al., 2019; Liang et al., 2015) were fully conserved (Table 1).

The M, P, and F proteins cooperate in the assembly of virus-like particles (VLPs) (Meshram et al., 2016; Mandviwala et al., 2025). Critical regions on the P protein required for interaction with M (aa 98–109, 115–125, and 131–151) remained unchanged (Bajorek et al., 2021). One ON1 strain exhibited an E49D substitution within the 39–57 region of P, which is necessary for VLP formation and known to require dephosphorylation (Meshram and Oomens, 2019); however, this mutation is unlikely to affect phosphorylation status. Mutations in the M protein included N27D and M73L within the N-terminal region (aa 1–200), which mediates self-interaction with the C-terminal domain (Trevisan et al., 2018). Phosphorylation sites S95 and T205, essential for M-mediated nuclear transport (Ghildyal et al., 2022), were conserved across all ON1 strains (Table 1).

For the 11 RSV-B BA9 strains, comparison with the early Chinese reference strain RSVB/BCH-Y/2016 revealed amino acid changes in NS1, NS2, N, P, SH, G, F, M2-1, M2-2, and L proteins. The CX3C motif in the G protein remained conserved. Within the key G protein antigenic region (aa 153–197), N176S (2 strains) and S189N (1 strain) substitutions were detected. Additionally, 25 mutations and a Q311 premature stop codon were observed in the C-terminal region.

In the F protein, all 11 strains harbored I206M and Q209R mutations within site Ø, while six strains carried an S389P substitution in site I. All strains exhibited a K191R substitution, and six strains carried S190N mutations in site V. Within the p27 domain, H114Y was consistently observed, while L125P was present in two strains (Table 2).

**Table 2:** Amino acid substitutions in RSV-B proteins among 11 BA9 strains from Jining compared to the early Chinese BA9 reference strain RSVB/BCH-Y/2016.

Protein	Amino acid substitution sites compared with RSVB/BCH-Y/2016
NS1	H67P (100%), E74K (100%), W92L (100%)
NS2	T3I (9.09%), D6N (18.18%), T28I (18.18%)
N	I303V (18.18%), N380H (100%)
P	T92A (9.09%)
M	–
SH	I32V (9.09%), I40L (18.18%), I40V (9.09%), I49L (27.27%), Y61H (9.09%), I63T (9.09%), N64D (100%)
G	A42V (18.18%), V45I (27.27%), I87T (18.18%), R98M (27.27%), V99A (9.09%), S100G (54.55%), P101L (9.09%), A107T (9.09%), A116T (18.18%), I118T (18.18%), N121S (18.18%), T126P (9.09%), A131T (45.45%), T137I (45.45%), S138A (9.09%), P140S (18.18%), P150T (9.09%), R151H (9.09%), N176S (18.18%), S189N (9.09%), Y213D (100%), P214S (54.55%), P217L (100%), P221L (54.55%), I227T (18.18%), K231E (18.18%), K232E (9.09%), L250F (9.09%), D251N (18.18%), I252T (63.64%), T253I (9.09%), T254I (18.18%), K256N (45.45%), T264N (9.09%), S265L (9.09%), S265P (18.18%), I268T (63.64%), V269A (54.55%), T274A (18.18%), S275P (54.55%), P284L (100%), Y285H (63.64%), T288I (100%), P289S (9.09%), N294Y (9.09%), N294H (18.18%), S295F (9.09%), T300I (18.18%), P301A (100%), S309F (9.09%), T310I (100%), Q311L (9.09%), Q311stop (90.91%)
F	F12I (18.18%), R42K (18.18%), H114Y (100%), L125P (18.18%), S190N (54.55%), K191R (100%), I206M (100%), Q209R (100%), S211N (54.55%), S389P (54.55%), L467F (18.18%), T529A (100%)
M2-1	I104T (18.18%), A160T (100%)
M2-2	M27T (54.55%), M27I (9.09%), D35N (54.55%), C49F (54.55%), C49R (9.09%), P79L (18.18%), I85V (100%)
L	N8S (90.91%), L56I (100%), S166N (100%), S169P (45.45%), N172D (9.09%), Y177N (27.27%), I197M (100%), H568N (9.09%), K570R (54.55%), R702S (100%), K705N (100%), S876N (100%), S970N (9.09%), M973T (100%), N1247S (9.09%), S1250G (100%), T1381I (100%), V1479A (72.73%), K1547R (100%), V1646I (18.18%), I1651V (9.09%), H1707N (18.18%), T1712A (100%), I1716V (100%), P1720S (9.09%), S1726R (100%), N1727S (9.09%), S1734N (9.09%), N1736S (100%), T1737A (27.27%), T1737V (9.09%), I1740M (100%), M1742T (90.91%), R1759K (54.55%), K1764R (100%), N1773I (100%), A1787E (100%), I1794T (100%), T1882N (100%), A1943T (27.27%), V1965I (18.18%), F2030L (100%), K2041R (9.09%), T2042I (100%), K2065N (100%), T2069A (9.09%),

Mutations affecting the M2-1–P interaction interface included an A160T substitution in M2-1 (present in all strains) and a T92A mutation in the P protein (one strain), which may influence RNP complex assembly. All critical residues in the N protein for interaction with P and viral RNA were conserved. The RdRp, Mtase, and PRNTase catalytic residues in L and P–M interaction domains remained unchanged (Table 2).

NS1 and NS2 proteins suppress host type I interferon (IFN) responses. The α3 helix region (aa 119–139) of NS1, essential for IFN suppression (Merritt et al., 2023; Chatterjee et al., 2017), and NS2 residues 24–26 and 32–33, known to inhibit IFN-*β* expression (Pei et al., 2021), were conserved in all BA9 strains (Table 2).

### 3.6 Analysis of glycosylation and phosphorylation sites

N-linked glycosylation site prediction using NetNGlyc 1.0 revealed notable alterations in RSV-encoded proteins among strains circulating in Jining. In the RSV-A ON1 subset, 12 out of 18 strains acquired a novel glycosylation site at asparagine 299 (N299) of the G glycoprotein. This site resides within the immunologically critical 84–85 C-terminal region, and the acquisition of N299 glycosylation may potentially modulate epitope conformation and reduce recognition by host neutralizing antibodies. Among the 11 RSV-B BA9 strains, glycosylation changes were more diverse: two strains acquired an additional N69 glycosylation site in NS2; two gained N114, and five gained N256 glycosylation sites in the G protein. In contrast, loss of glycosylation sites was also observed, including N294 (in two strains), N308 (in all strains), and N167 in the L protein (five strains). Notably, the glycosylation sites N256, N294, and N308 in the G protein are located within or adjacent to the C-terminal antigenic domain and are implicated in modulating viral immune evasion (Table 3).

**Table 3:** Analysis of phosphorylation and N-glycosylation site variations in RSV proteins from Jining.

Protein	RSV-A vs. BJ/40180				RSV-B vs. RSVB/BCH-Y/2016			
	Phospho sites		N-Gly sites		Phospho sites		N-Gly sites	
	↑	↓	↑	↓	↑	↓	↑	↓
NS1	-	-	-	-			-	-
NS2	-	-	-	-	-	T3 (9.09%), T28 (18.18%)	NNT6-9 (18.18%)	-
N	S105 (11.11%)	S372 (100%)	-	-	-	-	-	-
P	-	T69 (5.56%), T72 (16.67%), T92 (94.44%)	-	-	-	T92 (9.09%)	-	-
M	-	-	-	-	-	-	-	-
SH	-	-	-	-	T63 (9.09%)	Y61 (9.09%)	-	-
G	T10 (5.56%), S95 (22.22%), T102 (11.11%), T133 (5.56%), S143 (5.56%), T230 (16.67%), S234 (5.56%), S284 (16.67%)	T12 (50.00%), T113 (94.44%), S124 (5.56%), S102 (11.11%), T219 (11.11%), T231 (11.11%), T241 (16.67%), S260 (5.56%), S283 (5.56%), S291 (5.56%), Y297 (5.56%), S299 (66.67%)	NPS299-301 (12)	-	T107 (9.09%), T116 (18.18%), T118 (18.18%), S121 (18.18%), T131 (45.45%), S140 (18.18%), T150 (9.09%), S176 (18.18%), S214 (54.55%), T227 (18.18%), T252 (63.64%), S289 (9.09%), Y294 (9.09%)	S100 (54.55%), T137 (45.45%), S189 (9.09%), T253 (9.09%), T254 (18.18%), T264 (9.09%), S265 (27.27%), T274 (18.18%), S275 (54.55%), Y285 (63.64%), T288 (100%), S295 (9.09%), T300 (18.18%), T310 (100%)	NST114-116 (18.18%)、NHT256-258 (45.45%)	NST294-296 (18.18%)、NST308-310 (100%)
F	T103 (11.11%), S121 (50.00%)	-	-	NNT120-122 (9.09%)	Y114 (100%)	S389 (54.55%)	-	-
M2-1	-	-	-	-	T104 (18.18%), T160 (100%)	-	-	-
M2-2	T60 (5.56%)	T77 (88.89%), S60 (5.56%),	-	-	-	-	-	-
L	S173 (16.67%), S1550 (22.22%), T1737 (50.00%)	S1723 (5.56%), S1745 (5.56%), T1942 (5.56%), T1969 (5.56%)	-	-	N8S (90.91%), R702S (100%), P1720S (9.09%), N1727S (9.09%), I1794T (100%)	S169 (45.45%), Y177 (27.27%), S1250 (100%), T1381 (100%), S1726 (100%), T2042 (100%), T2069 (9.09%)	-	NQS167-169 (45.45%)

↑ = increased (gained), ↓ = decreased (lost); Phospho Sites = predicted phosphorylation sites; N-Gly Sites = predicted N-linked glycosylation sites.

Phosphorylation site prediction using NetPhos 3.1 identified several substitutions that may alter protein phosphorylation patterns, particularly within known antigenic regions. In RSV-A ON1 strains, three exhibited a gain-of-function S284 phosphorylation site in the G protein, while phosphorylation potential at T241 (3 strains), S260, S283, S291, Y297, and S299 (12 strains) was reduced. All these residues map to the immunodominant C-terminal epitope region of the G protein, suggesting a potential role in immune modulation. In the F glycoprotein, nine strains exhibited an additional S121 phosphorylation site within the p27 domain, which is critical for membrane fusion. Moreover, 11 ON1 strains demonstrated loss of T92 phosphorylation in the P protein, a residue located within the P protein’s 90–110 domain, which mediates interaction with M2-1 (Table 3).

In RSV-B BA9 strains, two sequences showed a novel phosphorylation site at S176 within the G protein’s 153–197 antigenic epitope. Additionally, phosphorylation gain was noted at T252 (7 strains), S289 (1 strain), and Y294 (1 strain), all localized within the C-terminal epitope region. Conversely, a substantial reduction in phosphorylation potential was observed at multiple sites within this region: S189, T253, T254, T264, S265, T274, S275, Y285, T288, S295, T300, and T310—several of which were conserved losses across most or all strains. These extensive modifications within the G protein’s antigenic domain underscore the dynamic evolution of post-translational modification (PTM) landscapes, potentially contributing to altered antigenicity and immune escape.

In the F glycoprotein of BA9 strains, a reduction in phosphorylation at S389—a site located within antigenic site I—was observed in six strains, which may impact antibody recognition or membrane fusion efficiency. All 11 strains displayed phosphorylation at Y114 within the p27 domain, further supporting the immunomodulatory relevance of this region. Furthermore, gain of phosphorylation at T160 in the M protein was detected in all BA9 strains. This residue is critical for M2-1 interaction with the P protein, suggesting that this PTM may influence RNP complex formation. Finally, one BA9 strain exhibited loss of T92 phosphorylation in the P protein, again located in the M2-1 interaction interface, and potentially affecting complex stability and viral transcription dynamics (Table 3).

## 4 Discussion

Viral infections remain a leading cause of morbidity and mortality worldwide, underscoring their persistent burden on global health systems and their impact on vulnerable populations (Chen X. et al., 2017; Duan et al., 2018; Chen Y. et al., 2017; Shi et al., 2017; Yuan et al., 2023; Li et al., 2016; Jiao et al., 2017; Li et al., 2025). RSV exhibits strong seasonality in China, with peak activity typically occurring in winter and spring, and occasional outbreaks in early summer in certain years (Luo et al., 2020; Jiang et al., 2025). The ON1 and BA9 genotypes currently represent the predominant circulating RSV lineages in China (Jiang et al., 2025; Li F. et al., 2024). The RSV positivity rate varies significantly across different years and regions in China. From July 2021 to January 2022, the positivity rate reached 21.79% among 1,225 samples in Hangzhou City; from January 1, 2024, to December 31, 2024, it was 4.8% among 5,373 samples in Hebei Province. In contrast, from January 2021 to December 2023, the positivity rate was only 1.81% among 125,409 samples in Shanghai (Guo et al., 2023; Lang et al., 2025). In this study, two distinct RSV epidemic peaks were identified in Jining between February 2023 and December 2024: one in April–May 2023 and another in December 2023–January 2024. These findings are consistent with prior reports indicating RSV circulation during both winter–spring and early summer periods in China, including a nationwide RSV surge in April–May 2023 (Li B. S. et al., 2024). The first peak was primarily caused by RSV-A, while the second was dominated by RSV-B, reflecting the typical genotype alternation pattern previously observed across China (Luo et al., 2020). The higher positivity rate in children under five aligns with the known age distribution of RSV infection in China (Luo et al., 2020). Previous studies have reported inconsistent findings regarding sex differences in RSV positivity rates. In a study conducted in Hangzhou City, China, from July 2021 to January 2022, no statistically significant difference in RSV positivity was observed between males and females. In contrast, from January 2021 to December 2023 in Shanghai, China, the RSV positivity rate was higher in males than in females (Guo et al., 2023).

Our data revealed a significantly higher positivity rate in males in Jining during the study period, suggesting potential sex-related or strain-specific differences in local RSV transmission. Furthermore, research indicates that sex is a significant factor influencing the progression of RSV infection, with males experiencing more severe symptoms and a higher risk of hospitalization. Therefore, enhanced efforts should be directed toward RSV screening and prevention in males to reduce both the incidence and severity of RSV infection in this population.

A total of 29 complete RSV genomes were obtained, including 18 RSV-A ON1 and 11 RSV-B BA9 strains, consistent with national trends. The persistence of ON1 and BA9 as dominant genotypes underscores the importance of continued genomic surveillance and the urgent need for genotype-specific vaccine development. Notably, the RSV-A ON1 genomes in Jining were classified into two clades (A.D.3 and A.D.5.2), while the BA9 genomes were distributed among four clades (B.D.E.1, B.D.E.1.2, B.D.4.1.1, and B.D.E.2). These findings highlight the genetic complexity of RSV circulation in Jining, with multiple clade variants circulating within the same genotype. This demonstrates that RSV is continuously evolving. Therefore, enhanced RSV surveillance should be implemented to track genotype transmission chains, thereby providing a scientific basis for the early warning of emerging novel variants.

Among RSV genes, the G gene is known to be the most variable (Anderson et al., 2021; Johnson et al., 1987). Consistent with this, our analysis revealed the lowest nucleotide and amino acid similarity for the G gene among both ON1 and BA9 sequences, indicating continuous antigenic drift. Compared to early reference strains (BJ/40180 for ON1 and RSVB/BCH-Y/2016 for BA9), 29 amino acid substitutions were observed in the G protein antigenic regions of ON1 strains, and 27 substitutions plus one premature stop codon (Q311*) were detected in BA9 strains. In contrast, only a single antigenic mutation was found in the F protein of ON1 strains, while five were identified in BA9 strains. These G and F protein variations may alter epitope structure, impair host immune recognition, and contribute to reinfection.

The C-terminal region of the G protein in RSV-B BA9 strains is highly prone to mutation, with position Q311 being a recognized hotspot for variation. The Q311stop mutation results in the truncation of the G protein, leading to the loss of the C-terminal seven amino acids. Previous studies have shown that this seven-amino-acid segment in the C-terminus of RSV-B BA9 strains enhances viral replication efficiency and increases G protein virulence, resulting in significantly elevated expression of TNF-*α* and IL-6, higher viral loads in lung tissue, and intensified cytokine-mediated inflammatory responses (Guo et al., 2023; Haider et al., 2018).

In the present study, 10 of the 11 BA9 sequences harbored the Q311stop mutation. This mutation may lead to reduced viral replication efficiency and attenuated virulence, potentially diminishing the population transmissibility of RSV-B BA9 strains. This could be one of the contributing factors to the relatively low RSV positivity rate observed in Jining city from February 2023 to December 2024. These implications are supported by structural modeling of the BA9 G protein C-terminus truncation (Supplementary Figure 3) and F protein mutations (Supplementary Figure 4), which may affect antigenic sites. Linear maps highlight variation frequencies (Supplementary Figures 1, 2).

The p27 domain of the F protein plays a critical role in membrane fusion. Four amino acid substitutions were detected in the p27 domain of ON1 strains, and two in BA9 strains, which may impact F protein function. In addition, the E239D mutation in the P protein (detected in one ON1 strain) maps to a critical interface for N protein binding. Similarly, the A160T mutation in M2-1 and T92A mutation in P—both detected in BA9 strains—are located within the P–M2-1 interaction domain and may affect ribonucleoprotein (RNP) complex stability. The E49D mutation in the P protein (ON1 strain) was located in the critical VLP-forming region (aa 39–57) and may influence particle assembly.

Post-translational modifications (PTMs), including glycosylation and phosphorylation, are essential for RSV protein function and immune evasion. N-linked glycosylation at residues such as N27, N70, N116, N126, and N500 on the F protein can alter immunogenicity and fusion activity (Tian et al., 2017; Jacobs et al., 2024; Leemans et al., 2019). The G protein contains multiple glycosylation sites that contribute to immune escape (Aziz et al., 2024; Gorman et al., 2001; Rawling and Melero, 2007; Garcia-Beato et al., 1996). In our study, N299 glycosylation was newly acquired in 12 ON1 G proteins, and N256, N294, and N308 glycosylation changes were found in BA9 strains—all located within G protein antigenic regions.

Phosphorylation also plays a pivotal role in regulating RSV replication and host interaction. The P protein contains more than 40 phosphorylation sites, including S54, T105, T108, S116, S117, S119, S143, S156, T160, T188, S203, T210, S232, and S237 (Asenjo et al., 2005; Mazumder et al., 1994; Asenjo et al., 2008b; Navarro et al., 1991). S54 phosphorylation regulates P–M binding and membrane translocation of RNP complexes (Asenjo et al., 2005; Asenjo et al., 2008a), while T108 is required for P–M2-1 complex formation (Asenjo et al., 2006). Phosphorylation at S143, T160, and T210 modulates transcriptional activity; S116, S117, S119, S232, and S237 are required for M2-2-mediated inhibition of viral RNA synthesis (Asenjo and Villanueva, 2016); and sites such as T105, T188, S203, and T210 are critical for viral replication (Beavis et al., 2021). The phosphorylation status of M protein residues S95 and T205 influences oligomerization and viral production (Ghildyal et al., 2022; Bajorek et al., 2014). M2-1 phosphorylation at S58 and S61 regulates RNA binding and transcriptional activity (Tanner et al., 2014; Cartee and Wertz, 2001). Moreover, phosphorylation changes at P and M2-1 interaction sites may impact RNP assembly and viral replication.

In conclusion, this study provides a comprehensive analysis of RSV epidemiology and genomic variation in Jining, China, from February 2023 to December 2024. The findings offer valuable insights into genotype distribution, clade diversity, and evolving antigenic features of RSV circulating in Jining. These results emphasize the need for continuous genomic surveillance, timely public health response, and the acceleration of genotype-specific RSV vaccine development to mitigate the impact of future outbreaks.

## Data Availability

The original contributions presented in the study are included in the article/Supplementary material, further inquiries can be directed to the corresponding authors.

## Footnotes

1
https://clades.nextstrain.org/

## Supplementary material

SUPPLEMENTARY FIGURE 1 Linear mutation map of amino acid variations in RSV-A proteins. Bar graph showing the percentage (%) of amino acid variations at each position across 18 RSV-A ON1 strains from Jining (2023–2024). Key mutations (e.g., S372A, T92M, N27D, H266L) are labeled. X-axis: amino acid positions; y-axis: variation frequency. Focus on antigenic regions in G and F proteins.

SUPPLEMENTARY FIGURE 2 Linear mutation map of amino acid variations in RSV-B proteins. Bar graph illustrating variation frequency (%) at each position in 11 RSV-B BA9 strains from Jining (2023–2024). Annotated mutations include H67P, E74K, Q311stop, S389P. X-axis: AA positions; y-axis: variation %. Emphasizes hypervariable sites in G and F proteins.

SUPPLEMENTARY FIGURE 3 Predicted 3D structure of the BA9 genotype G protein and C-terminal region (residues 311–317). Ribbon diagram showing the G protein structure with a close-up of the C-terminus (residues Q311, R312, L313, Q314, S315, Y316, A317 labeled). The Q311stop mutation (red arrow) causes truncation of seven amino acids, potentially reducing virulence. Predicted using AlphaFold and visualized in PyMOL (colors: blue for helices, orange for loops).

SUPPLEMENTARY FIGURE 4 Predicted 3D structure of the BA9 genotype F protein with key mutation sites (K191R, I206M, Q209R, S389P, S190N). **(A)** Overall trimeric F protein structure with mutations highlighted (S389P, S190N labeled). **(B)** Zoomed views of K191R, I206M, Q209R. These sites are near antigenic epitopes and may impact vaccine efficacy. Modeled based on PDB: 5C69 (pre-fusion F) using PyMOL.
