# Supplementary material for: Epidemiological characteristics and whole-genome analysis of respiratory syncytial virus in Jining city from February 2023 to December 2024
Source: Front Microbiol. 2026 Feb 11;17:1702525. doi: 10.3389/fmicb.2026.1702525 (PMC12932593; doi:10.3389/fmicb.2026.1702525)
Supplement: Supplementary file 5 [file Table_1.docx]

**Supplementary Table 1. Summary of RSV Detection Results in Jining (February 2023 – December 2024)**

|  | Sample mumber | RSV | |  | RSV-A | RSV-B | RSV-A+B |
| --- | --- | --- | --- | --- | --- | --- | --- |
|  |  | Positives number | Positives rate(%) |  | Positives number | Positives number | Positives number |
| Age（in years） |  |  |  |  |  |  |  |
| ＜1 | 56 | 3 | 5.34 |  | 1 | 2 | 0 |
| ≥1-＜5 | 1009 | 59 | 5.85 |  | 44 | 13 | 2 |
| ≥5-＜15 | 1217 | 21 | 1.73 |  | 16 | 5 | 0 |
| ≥15-＜25 | 702 | 2 | 0.28 |  | 1 | 1 | 0 |
| ≥25-＜60 | 1437 | 9 | 0.63 |  | 4 | 5 | 0 |
| ≥60 | 621 | 6 | 0.97 |  | 0 | 6 | 0 |
|  |  |  |  |  |  |  |  |
| Gender |  |  |  |  |  |  |  |
| Males | 2485 | 60 | 2.41 |  | 41 | 19 | 0 |
| Females | 2557 | 40 | 1.56 |  | 25 | 13 | 2 |
|  |  |  |  |  |  |  |  |
| Inpatient/ outpatient |  |  |  |  |  |  |  |
| Outpatients | 4450 | 88 | 1.98 |  | 65 | 21 | 2 |
| Inpatients | 592 | 12 | 2.03 |  | 1 | 11 | 0 |
| Total | 5042 | 100 | 1.98 |  | 66 | 32 | 2 |
