# Supplementary material for: Epidemiological characteristics and whole-genome analysis of respiratory syncytial virus in Jining city from February 2023 to December 2024
Source: Front Microbiol. 2026 Feb 11;17:1702525. doi: 10.3389/fmicb.2026.1702525 (PMC12932593; doi:10.3389/fmicb.2026.1702525)
Supplement: Supplementary file 6 [file Table_2.docx]

**Supplementary Table 2. Monthly RSV Positivity Rates in Jining (February 2023 – December 2024)**

| \| Month \| \| --- \| \|  \| | Sample mumber | RSV | | RSV-A | RSV-B | RSV-A+B |
| --- | --- | --- | --- | --- | --- | --- | --- | --- |
|  |  | Positives number | Positives rate(%) | Positives number | Positives number | Positives number |
| 2023-2 | 28 | 0 | 0.00 | 0 | 0 | 0 |
| 2023-3 | 270 | 1 | 0.37 | 1 | 0 | 0 |
| 2023-4 | 244 | 29 | 11.89 | 26 | 3 | 0 |
| 2023-5 | 297 | 40 | 13.47 | 34 | 4 | 2 |
| 2023-6 | 235 | 0 | 0.00 | 0 | 0 | 0 |
| 2023-7 | 265 | 0 | 0.00 | 0 | 0 | 0 |
| 2023-8 | 260 | 0 | 0.00 | 0 | 0 | 0 |
| 2023-9 | 249 | 0 | 0.00 | 0 | 0 | 0 |
| 2023-10 | 291 | 0 | 0.00 | 0 | 0 | 0 |
| 2023-11 | 250 | 0 | 0.00 | 0 | 0 | 0 |
| 2023-12 | 231 | 10 | 4.33 | 1 | 9 | 0 |
| 2024-1 | 283 | 15 | 5.30 | 3 | 12 | 0 |
| 2024-2 | 229 | 2 | 0.87 | 0 | 2 | 0 |
| 2024-3 | 220 | 1 | 0.45 | 0 | 1 | 0 |
| 2024-4 | 277 | 1 | 0.36 | 0 | 1 | 0 |
| 2024-5 | 218 | 0 | 0.00 | 0 | 0 | 0 |
| 2024-6 | 74 | 0 | 0.00 | 0 | 0 | 0 |
| 2024-7 | 67 | 0 | 0.00 | 0 | 0 | 0 |
| 2024-8 | 114 | 0 | 0.00 | 0 | 0 | 0 |
| 2024-9 | 195 | 0 | 0.00 | 0 | 0 | 0 |
| 2024-10 | 218 | 0 | 0.00 | 0 | 0 | 0 |
| 2024-11 | 235 | 0 | 0.00 | 0 | 0 | 0 |
| 2024-12 | 292 | 1 | 0.34 | 1 | 0 | 0 |
| Total | 5042 | 100 | 1.98 |  |  |  |
