# Supplementary material for: Epidemiological characteristics and whole-genome analysis of respiratory syncytial virus in Jining city from February 2023 to December 2024
Source: Front Microbiol. 2026 Feb 11;17:1702525. doi: 10.3389/fmicb.2026.1702525 (PMC12932593; doi:10.3389/fmicb.2026.1702525)
Supplement: Supplementary file 7 [file Table_3.docx]

**Supplementary Table 3. Sequence Similarity Analysis of 18 RSV-A ON1 Strains from Jining**

| Gene | | Similarity Among the 18 Jining ON1 Strains (%) | | Similarity Between Jining ON1 Strains and Early Chinese ON1 Reference Strain BJ/40180 (%) | |
| --- | --- | --- | --- | --- | --- |
| Gene name | Protein name | Nucleotide | Amino acid | Nucleotide | Amino acid |
| Complete Genome Sequence | | 98.33%-99.99% | - | 98.84%-99.07% | - |
| NS1 | NS1 | 98.81%-100% | 100% | 99.29%-100% | 100% |
| NS2 | NS2 | 98.67%-100% | 100% | 99.20%-100% | 100% |
| N | N | 98.56%-100% | 99.49%-100% | 98.81%-99.40% | 99.49%-99.74% |
| P | P | 98.62%-100% | 97.93%-100% | 99.04%-99.86% | 98.76%-99.59% |
| M | M | 98.70-100% | 99.61%-100% | 99.09%-99.48% | 99.22%-99.61% |
| SH | SH | 97.43%-100% | 100% | 97.95%-99.49% | 100% |
| G | G | 94.61%-100% | 90.13%-100% | 96.41%-97.04% | 93.31%-96.18% |
| F | F | 98.61-100% | 98.95%-100% | 99.07%-99.48% | 99.48%-99.83% |
| M2 | M2-1 | 97.94%-100% | 98.97%-100% | 98.67%-99.64% | 99.48%-100% |
|  | M2-2 |  | 96.59%-100% |  | 97.73%-100% |
| L | L | 98.97%-100% | 99.48%-100% | 99.19%-99.37% | 99.62%-99.90% |
