# Supplementary material for: Epidemiological characteristics and whole-genome analysis of respiratory syncytial virus in Jining city from February 2023 to December 2024
Source: Front Microbiol. 2026 Feb 11;17:1702525. doi: 10.3389/fmicb.2026.1702525 (PMC12932593; doi:10.3389/fmicb.2026.1702525)
Supplement: Supplementary file 8 [file Table_4.docx]

**Supplementary Table 4. Sequence Similarity Analysis of 11 RSV-B BA9 Strains from Jining**

| Gene | | Similarity Among the 11 Jining BA9 Strains (%) | | Similarity Between Jining BA9 Strains and Early Chinese BA9 Reference Strain RSVB/BCH-Y/2016 (%) | |
| --- | --- | --- | --- | --- | --- |
| Gene name | Protein name | Nucleotide | Amino acid | Nucleotide | Amino acid |
| Complete Genome Sequence | | 98.46%-99.98% | - | 97.92%-98.18% | - |
| NS1 | NS1 | 99.52%-100% | 100% | 98.81%-99.05% | 97.84% |
| NS2 | NS2 | 98.24%-100% | 100% | 98.59%-99.65% | 100% |
| N | N | 98.46%-100% | 99.69%-100% | 98.77%-99.49% | 99.69%-100% |
| P | P | 98.71%-100% | 99.35%-100% | 98.71%-99.35% | 99.35%-100% |
| M | M | 98.83%-100% | 100% | 98.83%-99.22% | 100% |
| SH | SH | 96.97%-100% | 95.38%-100% | 97.98%-98.99% | 95.38%-98.46% |
| G | G | 95.70%-100% | 91.77%-100% | 96.02%-97.69% | 93.35%-95.89% |
| F | F | 98.43%-100% | 98.95%-100% | 97.97%-98.49% | 98.26%-99.13% |
| M2 | M2-1 | 98.55%-100% | 99.49%-100% | 98.79%-99.39% | 98.97%-99.49% |
|  | M2-2 |  | 95.56%-100% |  | 95.56%-98.89% |
| L | L | 98.78%-99.98% | 99.53%-100% | 97.82%-98.18% | 98.73%-98.92% |
