# Supplementary material for: Epidemiological characteristics and whole-genome analysis of respiratory syncytial virus in Jining city from February 2023 to December 2024
Source: Front Microbiol. 2026 Feb 11;17:1702525. doi: 10.3389/fmicb.2026.1702525 (PMC12932593; doi:10.3389/fmicb.2026.1702525)
Supplement: Supplementary file 9 [file Table_5.docx]

**Supplementary Table 5. Distribution of G-Clades and Whole-Genome Clades Among RSV-A and RSV-B Strains from Jining**

| RSV | G-Clade/Clades | Clade Name | Number of Strains | Proportion (%) |
| --- | --- | --- | --- | --- |
| RSV-A | G-Clade | ON1 | 18 | 100% |
|  | Clades | A.D.3 | 17 | 94.44% |
|  |  | A.D.5.2 | 1 | 5.56% |
| RSV-B | G-Clade | BA9 | 11 | 100% |
|  | Clades | B.D.E.1 | 4 | 36.36% |
|  |  | B.D.E.1.2 | 2 | 18.18% |
|  |  | B.D.4.1.1 | 3 | 27.27% |
|  |  | B.D.E.2 | 2 | 18.18% |
